# Formation of intramolecular dimer radical ions of diphenyl sulfones

**DOI:** 10.1038/s41598-020-76907-4

**Published:** 2020-11-13

**Authors:** Kazumasa Okamoto, Shunpei Kawai, Takahiro Kozawa

**Affiliations:** 1 The Institute of Scientific Research (ISIR), Osaka University, 8-1 Mihogaoka, Ibaraki, Osaka 567-0047 Japan; 2 Artificial Intelligence Research Center (AIRC-ISIR), ISIR, Osaka University, 8-1 Mihogaoka, Ibaraki, Osaka 567-0047 Japan; 3 Faculty of Engineering, Hokkaido University, Sapporo, Hokkaido 060-0005 Japan

**Keywords:** Physical chemistry, Chemical physics, Reaction kinetics and dynamics, Optical spectroscopy

## Abstract

Dimer radical ions of aromatic molecules in which excess charge is localized in a pair of rings have been extensively investigated. While dimer radical cations of aromatics have been previously produced in the condensed phase, the number of molecules that form dimer anions is very limited. In this study, we report the formation of intramolecular dimer radical ions (cations and anions) of diphenyl sulfone derivatives (DPs) by electron beam pulse radiolysis in the liquid phase at room temperature. The density functional theory (DFT) calculations also showed the formation of the dimer radical ions. The torsion barrier of the phenyl ring of DPs was also calculated. It was found that the dimer radical ions show the larger barrier than the neutral state. Finally, stability of the dimer radical anion is dependent on not only the inductive effect of the sulfonyl group but the conjugation involving the d-orbital of the S atom and the phenyl rings.

## Introduction

The dynamics of excess charges on organic molecules is important for charge transfer in photo-, electro-, and radiation-induced phenomena. Previously, this has attracted attention for applications including devices^1,2^ and biomolecules^3,4^. Dimer radical ions (cations and anions) of aromatic molecules are species in which one hole or electron is localized in a pair of aromatic rings in a stabilized form. Intermolecular dimer radical ions are produced through the interaction between monomer radical ions and neutral molecules. Intramolecular dimer radical ions are produced through molecular relaxation after neutral molecules are changed into radical ions. Previous reports have investigated the inter- and intra-molecular production of many dimer radical cations^5–13^ and trimer radical cations^14^ in the condensed phase, attributed to the charge resonance (CR) in plural aromatic rings. However, in many cases, dimer radical anions are not observed even if dimer radical cations are formed^8^. Dimer radical anions of aromatic molecules have been mainly formed in rigid matrixes^15–20^. However, some recent studies have reported the formation of dimer radical anions of some limited molecules in the liquid phase at room temperature (RT)^21–26^. The dimer radical anions observed at RT are classified into two types. One is the σ-type, which is formed between the radical anion of phenylethynyl compounds and the neutral molecule ^25,26^. The other is the π-type, in which phenyl rings with electron-withdrawing groups, such as CN- and F- substituents, overlap^21–24^. This is similar to the *π–π* stacking in dimer radical cations.

In recent years, sulfone was introduced as an electron-accepting building block in luminescent materials for organic light-emitting diodes (OLEDs)^27–35^. The structure of diphenyl sulfone has also been applied^36–41^. Furthermore, in an earlier study, we used DPs as additives in resist materials for electron beam (EB) and extreme ultraviolet lithography processes^42^. We found that adding DPs enhanced the resist sensitivity. Radical anions of DPs play an important role in transferring electrons to photoacid generators. Therefore, elucidating the dynamics of the radical anion of sulfone is important for the development of sulfone-based functional materials.

In this article, we report the formation of dimer radical ions of DPs in the liquid phase at room temperature. For the study, we used the DPs diphenyl sulfone and 4,4′-substituted (tolyl and methoxy) derivatives. An EB pulse radiolysis method was used to observe the radical ions, and computational calculations using density functional theory (DFT) were performed for comparison. We also discuss the factors contributing to the robustness of the dimer radical anion of diphenyl sulfones.

## Results and discussion

### Formation of the radical cation of DPs by pulse radiolysis

The pulse radiolysis method is a pump-probe method that can be used to observe short-lived intermediates after EB pulse irradiation. Time-resolved photoabsorption of intermediates formed by ionizing radiation are measured. In pulse radiolysis, radical ions (cations or/and anions) can be selectively formed by changing the solvent. To produce solute radical cations, halogenated hydrocarbons are frequently used as a solvent. To observe the dynamics of DP radical cations, we performed pulse radiolysis of the DP solution in 1,2-dichloroethane (DCE). In DCE, the irradiation of EB pulses induces the following reactions:

1 $$  {\text{DCE}}{ \rightsquigarrow }{\text{DCE}}^{{\boldsymbol \cdot} + } + {\text{ e}}^{-} , $$

2 $$ {\text{DCE}}^{{\boldsymbol \cdot} + } + {\text{So}}l \, \to {\text{Sol}}^{{\boldsymbol \cdot} + } + {\text{e}}^{-} , $$

3 $$ {\text{DCE }} + {\text{e}}^{-} \to {\text{ DCE}}^{{{\boldsymbol \cdot} {-}}} /{\text{ CH}}_{2} {\text{ClCH}}_{2}^{ \cdot } + {\text{ Cl}}^{-} . $$

First, the solvent (DCE) undergoes ionization and produces the DCE radical cation (DCE^**·**+^) and electrons (e^−^) (Eq. 1)^43^. DCE^**·**+^ shows absorption maxima at 360 and 550 nm^43^. DCE^**·**+^ transfers holes to solutes (Sol) with lower ionization potentials (Eq. 2)^44^ DCE scavenges electrons, producing DCE radical anions (DCE^**·**–^) or causing dissociative electron attachment (formation of a neutral radical (CH_2_ClCH_2_·) and a Cl anion (Cl^–^)) (Eq. 3).

**Figure 1:**
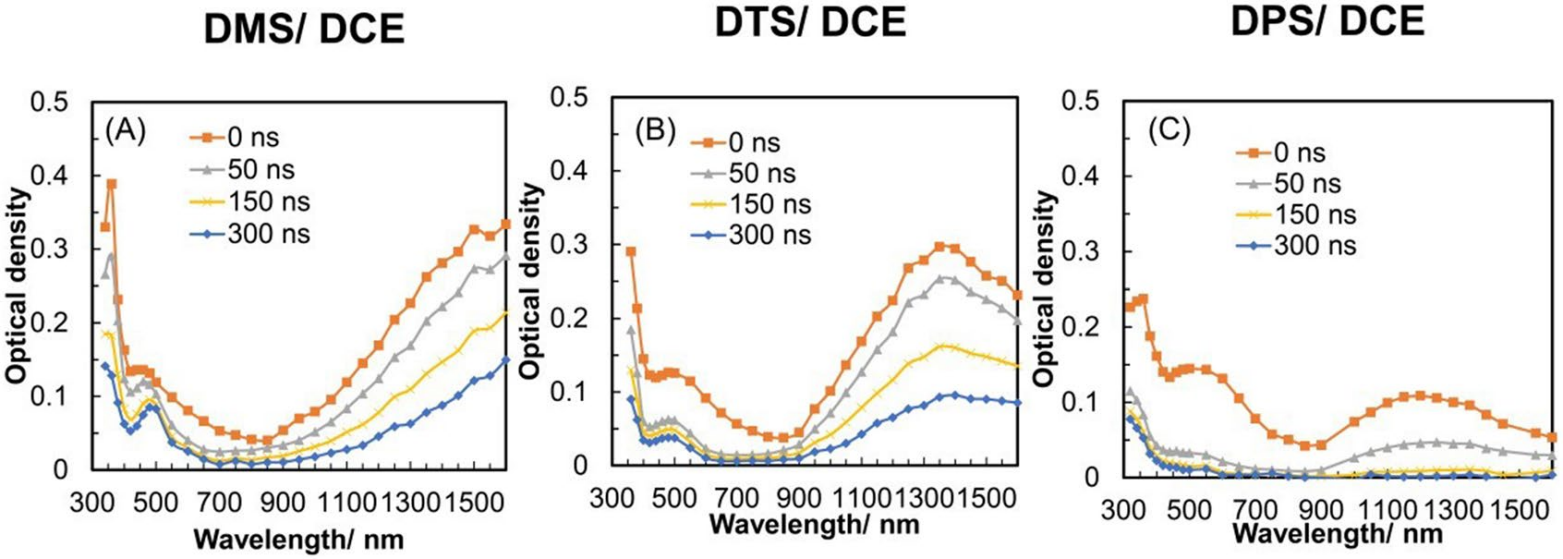
Transient absorption spectra of diphenyl sulfone derivative (DP) solutions (20 mM) in Ar-saturated 1,2-dichloroethane (DCE) obtained by the nanosecond pulse radiolysis method. The DPs were (**A**) 4,4′-dimethoxydiphenyl sulfone (DMS), (**B**) 4,4′-dimethyldiphenyl sulfone, and (**C**) diphenyl sulfone (DPS).

**Figure 2:**
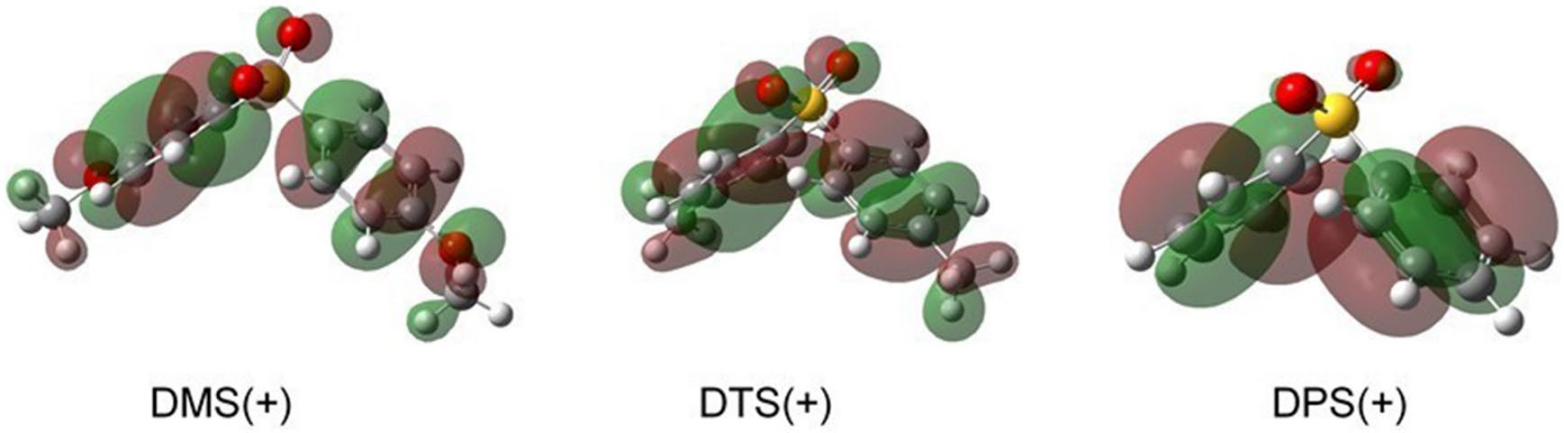
Optimized structures with the SOMO alpha orbitals (isovalue = 0.02) for the radical cations of DMS [DMS(+)], DTS [DTS(+)], and DPS [DPS(+)] calculated by the DFT method [B3LYP/6-31g + (d,p)].

Figure 1 shows the transient absorption spectra obtained by the pulse radiolysis of DPs solutions (20 mM) in DCE from the start of an 8 ns EB pulse (0 ns) to 300 ns after the pulse. At 0 ns, we could clearly observe the absorption bands of DCE^**·**+^ in diphenyl sulfone (DPS) and di-*p*-tolyl sulfone (DTS) solutions, which show absorption maxima at 360 and 500 nm, as reported previously^43^. These DCE^**·**+^ absorption bands in 4,4′-dimethoxydiphenyl sulfone (DMS) solution are not clear; we assume that the electron-donating methoxy group lowers the ionization potential of DMS and causes the fast hole transfer from DCE^**·**+^ to DMS during the 8 ns EB pulse. The characteristic near-infrared (NIR) absorption bands appeared at 0 ns in all DPs solutions, with absorption maxima at > 1600 nm (DMS), 1350 nm (DTS), and 1200 nm (DPS). The absorption intensity and the stability of the radical cations increased with the existence of electron-donating groups in the phenyl rings. The decay rate constants of the NIR bands were 5.5 × 10^6^ s^–1^ (DMS), 6.4 × 10^6^ s^–1^ (DTS), and 2.0 × 10^7^ s^–1^ (DPS). These bands were assigned to the charge resonance (CR) band of intramolecular dimer radical cations, in which a single positive charge is delocalized in two phenyl rings^5,6^. Since the shape of the CR band did not change even when the concentration of DPs was reduced to 1 mM, the contribution of the intermolecular dimer radical cation on the CR band is small. The charge delocalization between the phenyl rings of DP radical cations are represented by the calculated structures of the DP radical cations and the SOMO alpha orbital (Fig. 2). We also observed that increased distances between the DPs phenyl rings corresponded to longer absorption wavelengths (Table 1). Because the phenyl rings of DPs are not in the same plane [the C–S–C angle is about 100° (Table 1)], we expect the *π*-electrons between the phenyl rings to less overlap. The CR bands shifted toward a longer wavelength when an electron donating group was present.

**Table 1:** Calculated parameters (phenyl ring distances, C–S distances, dipole moments, and C–S–C angle) of DPs [neutral, radical cations (+), and radical anions (–)] calculated by DFT methods [B3LYP/6-31 + g(d,p)].

Calculated parameters	DPS			DTS			DMS		
	Neutral	+	–	Neutral	+	–	Neutral	+	–
Phenyl rings distances (nm)*	0.506	0.499	0.486	0.508	0.500	0.487	0.509	0.504	0.485
C–S distances (nm)	0.181	0.180	0.177	0.180	0.180	0.177	0.180	0.179	0.177
Dipole moment (debye)	5.61	7.72	4.22	6.28	8.19	5.81	7.09	8.95	7.95
C–S–C angle (deg)	104.9	102.4	108.5	105.2	101.9	108.6	105.8	102.9	109.6

*Distances between centers of phenyl rings.

According to the TD-DFT calculations, the absorption bands in NIR were predicted to be mainly due to the CR band (HOMO-1 → SOMO). We calculated the peak wavelength of the CR band using TD-DFT calculations, which included the polarizable continuum model (PCM) to represent the solvation effect; these results were consistent with the absorption maxima of CR bands obtained by the pulse radiolysis (Fig. S1). However, the difference between the experimental and calculated values tended to increase with increasing sulfone polarity. Especially in DMS, the orientation of each DCE molecule may influence the energy level of SOMO and HOMO-1 in the radical cations. The radical cation of 4,4′-diaminodiphenyl sulfone has been represented by a positive-charge localized model on one side of the phenyl ring due to shielding by a sulfonyl group, according to a computational calculation using the BHandHLYP functional^45^. However, our observations of the CR band suggest the dimer radical cation model of DPs.

Local excitation (LE) bands (SOMO → LUMO) were also observed in the transient absorption spectra of DPs solutions. Absorption maxima or shoulders could also be observed by pulse radiolysis at around 300 nm (Fig. 1). These absorptions were calculated using TD-DFT for the radical cation (oscillator strength (*f*) > 0.05) (Fig. S1). During the pulse radiolysis experiments, characteristic absorption maxima could also be observed at 500 nm 50 ns after EB irradiation in the DMS and DTS solutions. In the DPS solution, the band intensity is less obvious, similar to the CR band. The absorption at around 500 nm did not appear in the TD-DFT calculation for the radical cation (Fig. S1). Thus, we assumed that the absorption at 500 nm could be attributed to transient species that were not radical cations. Previous reports have shown that aromatics^46,47^ and sulfur-based molecules such as dimethyl sulfide and diphenyl sulfide^48,49^ produce a complex with halogen atoms during pulse radiolysis in chlorinated hydrocarbon solvents. These experiments have reported absorption maxima at around 500 nm. The formation of the complex can be mainly attributed to the reactions between solute radical cations and Cl^–^ derived from the dissociative attachment of chlorinated hydrocarbons. Therefore, DPs are also assumed to form a complex with Cl atoms (DPs-Cl·) in the following reaction:

4 $$ {\text{DPs}}^{{\boldsymbol \cdot} + } + {\text{Cl}}^{-} \to {\text{ DPs}} - {\text{Cl}}^{ \cdot } . $$

The yield of DPs-Cl· depends on the yield of DPs^**·**+^. This is consistent with the transient absorption behavior observed by pulse radiolysis.

### Formation of radical anions by pulse radiolysis

Solute radical anions are also produced by pulse radiolysis in tetrahydrofuran (THF). The reactions yielding the radical anion are summarized in the following equations^50,51^:

5 $$ {\text{THF}}{ \rightsquigarrow }{\text{THF}}^{{\boldsymbol \cdot} + } + {\text{e}}^{-} , $$

6 $$ {\text{THF}}^{ {\boldsymbol \cdot} + } + {\text{ THF}} \to {\text{ THF}}( - {\text{H}})^{ \cdot } + {\text{ THF}}({\text{H}}^{ + } ), $$

7 $$ {\text{Sol }} + {\text{e}}^{-} \to {\text{ Sol}}^{{{\boldsymbol \cdot} {-}}} . $$

First, THF is ionized to induce the ejection of electrons (Eq. 4). During the EB pulse, the THF radical cation (THF^**·**+^) causes extremely fast, exothermic deprotonation between THF molecules (Eq. 5), and the hole transfer from THF^**·**+^ to the solute is negligible. THF electron-acceptable solutes scavenge the solvated electrons, producing the radical anion (Sol^**·**–^). Since sulfones easily react with electrons, these reactions generate DP radical anions.

**Figure 3:**
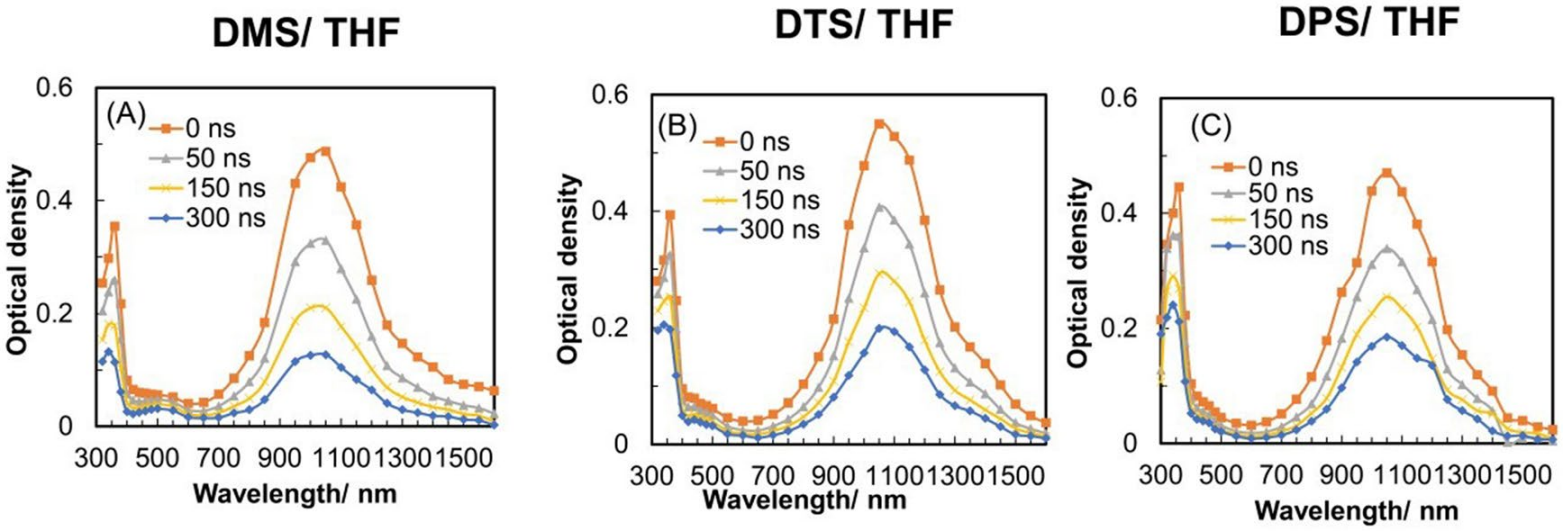
Transient absorption spectra of diphenyl sulfone derivative (DP) solutions (20 mM) in Ar-saturated 1,2-dichloroethane (DCE) obtained by the nanosecond-pulse radiolysis method. The DPs were (**A**) DMS, (**B**) DTS, and (**C**) DPS.

**Figure 4:**
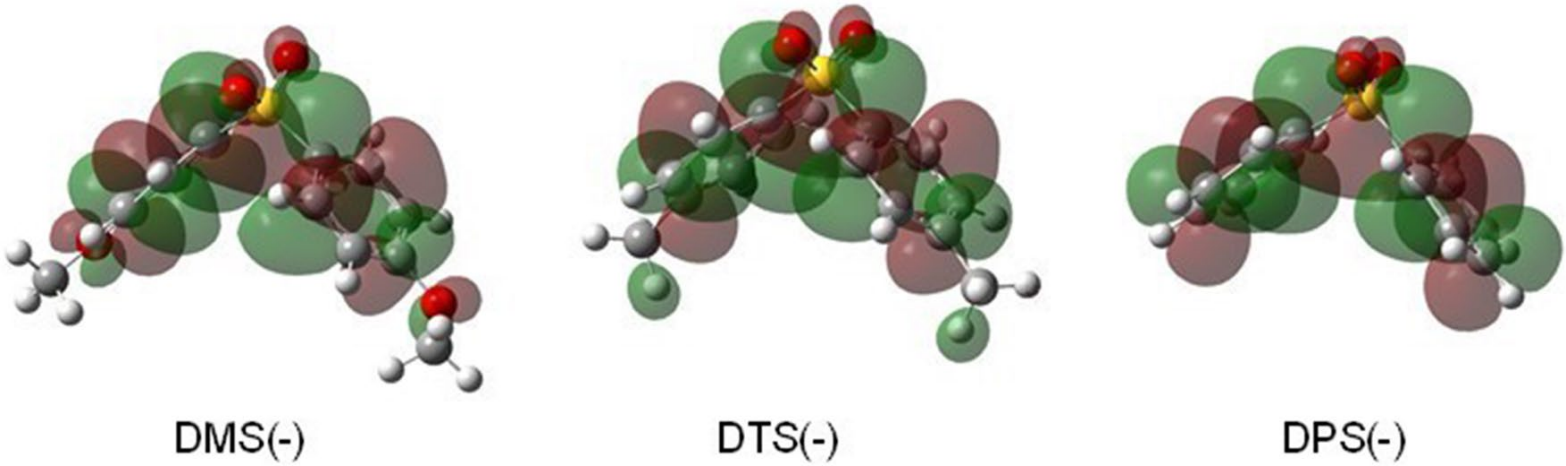
Optimized structures with the SOMO alpha orbitals (isovalue = 0.02) for the radical anions of DMS [DMS(–)], DTS [DTS(–)], and DPS [DPS(–)], calculated by the DFT method [B3LYP/6-31g + (d,p)].

Figure 3 shows the transient absorption spectra obtained from the pulse radiolysis of DP solutions (20 mM) in THF. Two absorption maxima, with sharp bands at 360 nm and broad bands at 1050 nm, are shown. Previously, similar absorptions in γ-irradiated methyltetrahydrofuran at 77 K were reported^52^. Unlike the DPs radical cations, the substituents do not affect the intensity of the absorption band. These absorption bands can be assigned to radical anions of DPs. To confirm these assignments, we compared the results with the DFT and TD-DFT calculations, as described above. The SOMO alpha orbital from the calculations shows charge delocalization through the phenyl rings and S atom (Fig. 4), indicating conjugation through the d-orbital of the S atom and π-electrons of the phenyl rings^52^. Previously, the spin densities of the radial anion of diphenyl sulfone were calculated using the Hückel calculation and compared with the result of ESR experiments^53,54^. However, previous studies did not consider the spin on the d-orbital of the S atom. As shown in Table 1, the S–C distances and the distances between the centers of the phenyl rings are shorter for the radical anions than for the radical cations and the neutral molecules. This suggests the effect of stabilization by conjugation despite the slightly larger C–S–C angle for the radical anion.

The absorption bands obtained by pulse radiolysis were also compared with the TD-DFT calculations to assign the transitions (Fig. S2). The TD-DFT calculations for the radical anions of DPs indicated transitions with high oscillator strength around 900 and 330 nm, which was consistent with the absorption maxima obtained by pulse radiolysis (Fig. 2). From the TD-DFT calculations, the NIR transition indicate the existence of the CR band (mainly SOMO → LUMO or SOMO → LUMO + n), while we predict the 330 nm band to be the LE band (HOMO-1 → SOMO). Therefore, the 360 and 1050 nm absorptions could be assigned to the LE band and CR band, respectively. The shape of the CR band shows no change even when the concentration of DPs was reduced to 1 mM. Hence, we suggest that intramolecular DP dimer radical anions formed at RT in THF.

The first-order decay rate constants of the DP dimer radical anion were calculated to be 7.3 × 10^6^ (DMS), 6.4 × 10^6^ (DTS), and 6.4 × 10^6^ (DPS) s^–1^. Generally, electron-donating groups destabilize the radical anion of aromatics. However, we did not observe a large substituent effect in the wavelength of the CR band absorption maxima. Since our absorption maxima for the CR bands were nearly identical to those in the low-temperature matrix^52^, we assumed a rigid structure for the DP radical anion. The structure of the radical anion is similar to that of the cation, and the π–π interaction between the phenyl rings is considered small. However, we assumed that the conjugation of the d-orbital of the S atom and the phenyl rings plays an important role in the rigidity. While the dimer radical anion is difficult to produce in liquid at RT, we conclude that this rigidity leads to the robustness of the DP radical anion.

### Rotation barrier of the phenyl ring of DP radical ions

**Figure 5:**
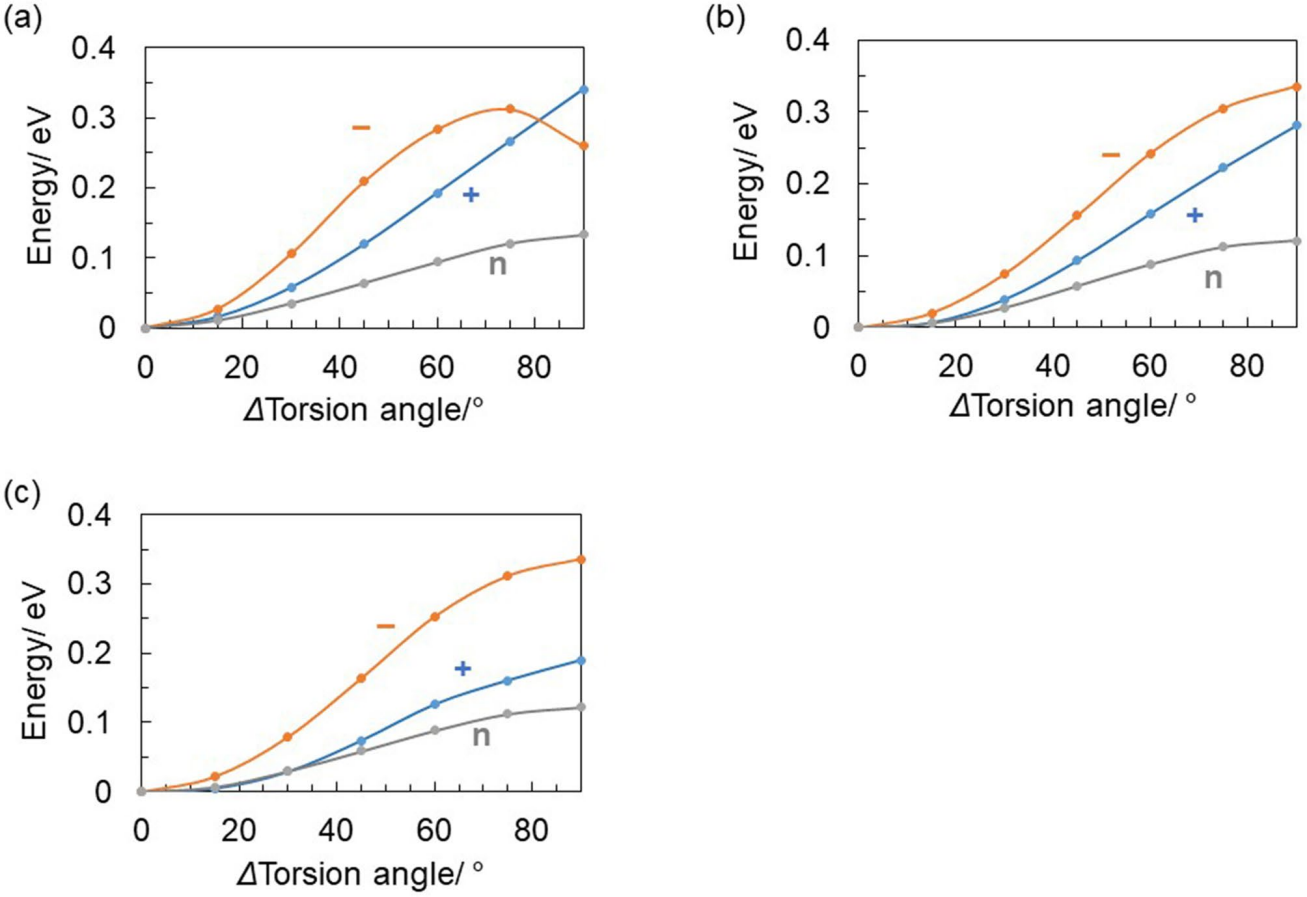
Torsion barriers of one phenyl ring in DPs from the optimized conformations of neutral states (n), radical cations (+), and radical anions (−) calculated by DFT methods [B3LYP/6-31 + g(d,p)] [(a) DMS, (b) DTS, and (c) DPS)]. All geometric parameters except for the torsion angle was fixed.

The rotation of the phenyl ring in DPs determines the rigidity and orientation of the phenyl rings. Therefore, investigating the rotation of the phenyl ring in DPs is helpful in discussing the stability of radical ions. For example, a previous study calculated that the energy barrier for torsion in the radical cation of biphenyl, calculated that in the radical cation of biphenyl, the torsion barrier of the phenyl rings was about 10 times larger than for the neutral state because of conjugation^55^. We calculated the torsion barriers of phenyl ring in the dimer radical ions and the neutral states of DPs using DFT. In the calculations, each optimization of DPs geometry was carried out with changing the torsion angle [(C_α_–S–C_α_–C_β_) C_α_: α-carbon, C_β_: β-carbon against sulfonyl group]) from the optimized structures. All geometric parameters except for the torsion angle was fixed according to the previous study^56^. The results are shown in Fig. 5. In the neutral state, the torsion barrier for the optimized structure of DPs was about 0.12–0.13 eV (2.8–3.0 kcal mol^–1^) at 90° in all cases. The radical ions had a larger torsion barrier than did the neutral states. The trend of torsion barrier for the radical cations was DMS > DTS > DPS, showing electron-donating substituent dependence. By contrast, the radical anion was nearly unaffected by the substituents and had nearly the same torsion barrier for all DPs except for the DMS around 90°. The minimum energy conformation of radical ions and neutral DMS is when the methoxy group lies in the plane of the phenyl rings. However, when the phenyl rings of the radical anion of DMS become perpendicular to each other (*Δ* Torsion angle ≈ 90°), the methoxy group attached to the phenyl ring fixed against the sulfonyl group lies in the perpendicular of the phenyl ring. Stabilization occurs due to changes in the orientation of the methoxy groups, and the radical anion does not show energy maximum around 90°.

Electron affinity (EA) of the radical anion of DPs is important factor for robustness of the radical anion. EA of the radical anion of DPs was also calculated using DFT [B3LYP/6-311 + G(d,p) ] between the differential energy of the neutral and the radical anion. The EA for DMS, DTS, DPS was 0.35, 0.13, 0.50 eV, respectively showing substituent dependence at the 4,4′-position of the phenyl ring. The inductive effect of the sulfonyl group in the diphenyl sulfone leads the positive electron affinity and contributes to the stability of the radical anion.

The contribution of conjugation in the d-orbital of the S atom in the radical anions enhanced the torsion barrier. The two phenyl rings of DPs are almost perpendicular, therefore, the π–π interactions through the face-to-face stacking hardly affect the stability of the dimer radical anions in DPs. Meanwhile, since the torsion barrier of radical ions were larger than that of neutral molecules, the stabilizing effects of the charge delocalization exist. We conclude that conjugation partially contribute to robustness of the radical anion of DPs. Similar charge delocalization through transannular interaction has been reported in radical anion of 1,1-diphenyl methane in γ-irradiated glassy matrix at 77 K^20^. Therefore, we found that the dimer radical anions of DPs produced in a liquid at RT are different from those previously reported, which were species with stacked phenyl rings and strong electron-withdrawing substituents^21–24^.

## Conclusions

To evaluate the formation of DP radical ions, we performed pulse radiolysis of DP solutions. As a result of transient absorption spectroscopy, CR bands from the dimer radical cations and anions could be observed in the NIR spectrum. The dimer radical ions displayed CR interactions without the face-to-face overlapping of phenyl rings of sulfone. The dimer radical cations experienced charge delocalization between the phenyl rings. In addition, we revealed that the dimer radical anion also showed excess charge delocalization on the phenyl rings and d-orbitals of the S atom. Not only the inductive effect of the sulfonyl group of DPs, but this conjugation induced the formation of the dimer radical anion and inhibited the rotation of the phenyl ring, enabling the formation of dimer radical anions even at RT. This radical ion intermediate could play an important role in applications such as organic conductive materials, as well as EUV and EB resists. Additionally, we conclude that the properties contribute important insights for the development of materials containing DPs.

## Methods

### Pulse radiolysis

Pulse radiolysis is a pump-and-probe method used to detect the short-lived intermediates produced by ionizing radiation. Pulse radiolysis was performed using the 26 meV L-band linear accelerator at the Institute of Scientific and Industrial Research (ISIR), Osaka University, as a pumping source and a Xe flash lamp as a probe light source. The pulse radiolysis had approximately 10 ns time resolution, and the pulse duration was 8 ns. The probe light was passed through a quartz sample cell simultaneously with the EB pulse and was sent to the monochromator and photodetectors [Si PIN-photodiode (λ = 350–950 nm) and InGaAs photodiode (λ = 950–1600 nm)]. The electrical signals (light intensity) were then sent to a digital oscilloscope, and a personal-computer. EB dosimetry was performed using a potassium thiocyanate (KSCN) dosimeter (5 mM KSCN in distilled water). The absorption dose per EB pulse was about 270 Gy.

Diphenyl sulfone (DPS) (97%, Sigma-Aldrich), di-*p*-tolyl sulfone (DTS) (99%, Sigma-Aldrich), and bis(4-methoxyphenyl sulfone) (DMS) (≥ 95%, Atomax chemicals) were used as solutes. Diphenyl sulfones (20 mM) were dissolved in tetrahydrofuran (≥ 99.9%, inhibitor-free, Sigma-Aldrich) and 1,2-dichloroethane (> 99%, Sigma-Aldrich). The sample solutions were saturated with Ar through a bubbling process in a quartz sample cell. The solutions were irradiated at RT.

The reaction rate constants were calculated and fitted to the kinetic traces using Origin Pro software (v. 2018b, OriginLab Corp.).

### DFT calculations

All calculations were performed using density functional theory (DFT). Optimized structures for the ground state, radical anion, and radical cation and their energies were obtained. The B3LYP functional and the 6-31 + G(d,p) basis set were used. TD-DFT calculations were also performed for radical anions and cations and were compared with the spectroscopic results from the pulse radiolysis. In the DFT and TD-DFT calculations, the polarizable continuum model (PCM) was used for the solvation effects. The torsion barriers of the phenyl ring of DPs (neutral molecules, radical cations, and radical anions) were also predicted using DFT. The energy was calculated without a solvation effect. All calculations were performed using the Gaussian09D program package^57^.

## Supplementary information

Supplementary Figures.
