# Supplementary material for: Formation of intramolecular dimer radical ions of diphenyl sulfones
Source: Sci Rep. 2020 Nov 13;10:19823. doi: 10.1038/s41598-020-76907-4 (PMC7666127; doi:10.1038/s41598-020-76907-4)
Supplement: Supplementary file 1 — Supplementary Figures. [file 41598_2020_76907_MOESM1_ESM.pdf]

Supplementary Information for

Formation of intramolecular dimer radical ions of diphenyl sulfones

Kazumasa Okamoto[1,2]*, Shunpei Kawai[3], Takahiro Kozawa[1]

1. The Institute of Scientific Research (ISIR), Osaka Univ., 8-1 Mihogaoka, Ibaraki, Osaka, Japan 567-0047

2. Artificial Intelligence Research Center (AIRC-ISIR), ISIR, Osaka Univ., 8-1 Mihogaoka, Ibaraki, Osaka, Japan 567-0047

3. Faculty of Engineering, Hokkaido Univ., Sapporo, Hokkaido, Japan 060-0005

*kazu@sanken.osaka-u.ac.jp (K. Okamoto)

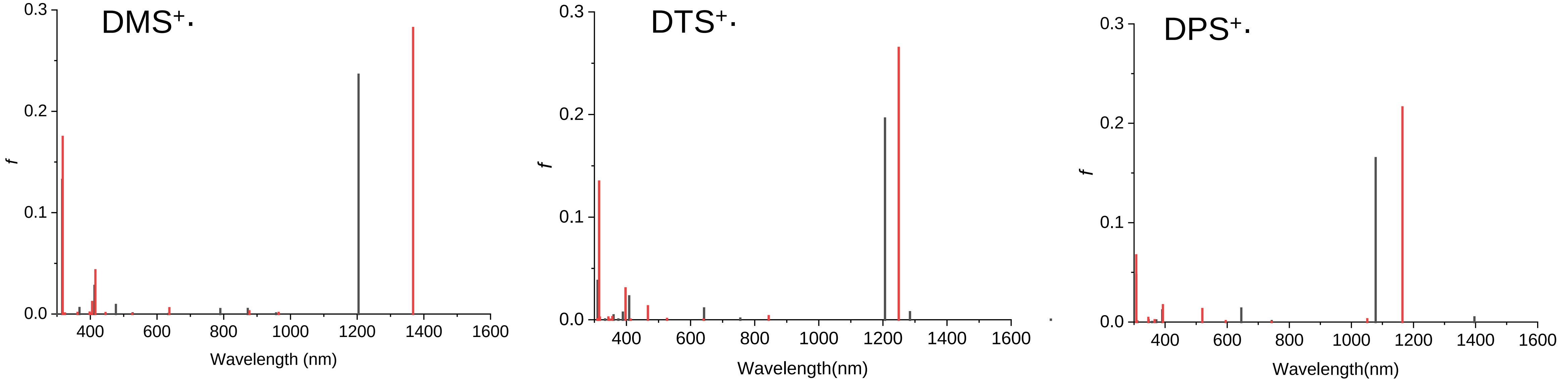

Fig. S1

Calculated excitation energy and oscillator strengths (*f*) for radical cations of diphenyl sulfones (DMS, DTS, and DPS) by TD-DFT method [B3LYP/6-311g+(d,p) // B3LYP/6-31g+(d,p)]. Black bars represent the calculated data without solvation effect and red bars include the polarizable continuum model (PCM) as a solvation effect in 1,2-dichloroethane.

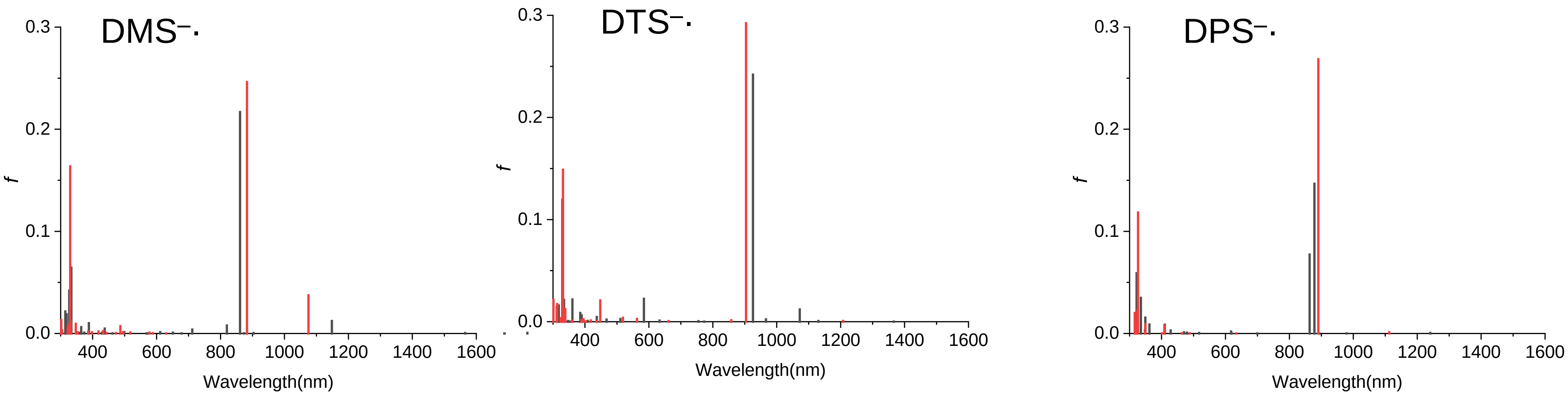

Fig. S2

Calculated excitation energy and oscillator strengths (*f*) for radical anions of diphenyl sulfones (DMS, DTS, and DPS) by TD-DFT method [B3LYP/6-311g+(d,p) // B3LYP/6-31g+(d,p)]. Black bars represent the calculated data without solvation effect and red bars include the polarizable continuum model (PCM) as a solvation effect in tetrahydrofuran.
